# Identification of tailored faculty development for diverse career trajectories in hospital medicine

**DOI:** 10.1002/jhm.70292

**Published:** 2026-03-13

**Authors:** Sachita Shrestha, Dave Bozaan, Shuo Tian, Stephanie Rennke, Lanna Felde, Maria Klimenko, Ashwini Niranjan‐Azadi, Pinky Jha, Elizabeth Murphy, David Paje, Stephanie Parks Taylor

**Affiliations:** ^1^ Department of Internal Medicine, Division of Hospital Medicine University of Michigan Ann Arbor Michigan USA; ^2^ Department of Medicine, Division of Hospital Medicine University of California‐San Francisco San Francisco California USA; ^3^ Department of Medicine, Division of Hospital Medicine The University of Texas Southwestern Medical Center Dallas Texas USA; ^4^ Division of Hospital Medicine University of Colorado School of Medicine Aurora Colorado USA; ^5^ Department of Medicine, Division of Hospital Medicine Johns Hopkins School of Medicine Baltimore Maryland USA; ^6^ Division of General Internal Medicine Medical College of Wisconsin Milwaukee Wisconsin USA; ^7^ Department of Medicine, Section of Hospital Medicine University of Chicago Medicine Chicago Illinois USA

## Abstract

**Background:**

Academic hospital medicine faculty possess diverse interests and pursue a variety of career pathways. Faculty development programs are essential for career advancement; however, a lack of alignment between individual faculty needs and development activities can impede career development efforts. To our knowledge, there are no studies that have examined whether clusters of faculty exist with similar development needs, for which tailored faculty development “packages” can be offered.

**Objective:**

To determine whether distinct clusters of faculty with shared development needs can be identified and to explore faculty‐level factors associated with these clusters.

**Method:**

We conducted a survey study among hospitalists at seven academic institutions from November 2023 to September 2024. We generated a comprehensive list of 33 potential faculty development topics and asked survey respondents to indicate whether each topic was important to them, choosing as many as applied. We used latent class analysis (LCA) to classify faculty into distinct clusters based on their selection of desired development topics.

**Results:**

A total of 136 faculty (median age 38 years) participated in the study. LCA identified five distinct groups as the optimal solution: (1) Master clinician (29%), (2) medical education scholar (11%), (3) clinical investigator (24%), (4) educational leader (22%), and (5) operational leader (14%).

**Conclusion:**

In a representative sample of academic hospitalists, we identified five distinct latent classes of faculty based on their preference for faculty development topics. These results can be leveraged to curate specific packages to optimally align faculty development offerings with diverse faculty interests and career trajectories.

## INTRODUCTION

Academic hospital medicine faculty members are critical to the healthcare delivery system and health professions education, with their roles encompassing patient care, teaching, research, and leadership.^1^, ^2^ Given the diverse range of roles that hospital medicine faculty fulfill, they also exhibit considerable diversity in professional interests, career trajectories, and development needs.^3^, ^4^ While faculty development programs are essential for supporting the academic, clinical, and leadership goals of hospitalists, one significant challenge lies in aligning these programs with the individual needs of each faculty member.^3^, ^5^, ^6^, ^7^ Many faculty members express dissatisfaction with the broad and often generic nature of existing development opportunities.^8^

To our knowledge, no studies have systematically examined whether clusters of faculty exist with similar development needs, which would allow tailoring of faculty development programs based on these identified clusters. The ability to offer targeted development “packages” that address the unique needs of specific groups within academic hospital medicine could enhance the relevance and effectiveness of these programs and potentially enhance community among faculty cohorts.

One such method for identifying subgroups or profiles is latent class analysis (LCA). LCA is a statistical method that allows clustering of distinct subgroups of a population that share similar characteristics based on patterns of responses to observed variables.^9^ Using LCA to identify subgroups of faculty with distinct needs may lead to more effective tailoring of faculty development programs that focus on combinations or packages of development activities. Integrated approaches can be developed that are cluster‐ rather than need‐specific, thereby taking into account their interrelatedness. The aim of this study was to identify which cluster profiles exist and to explore faculty‐level factors that are associated with different cluster profiles identified through LCA.

## METHODS

This was a multi‐institutional survey study across seven academic medical centers in the United States, targeting faculty members within hospital medicine divisions or adult hospitalists within the department of medicine. We generated a list of faculty development topics through an iterative and deliberate co‐design process. First, our study team started with an existing construct definition of faculty development from current literature: “any planned activity designed to improve an individual's knowledge and skills in areas considered essential to the performance of a faculty member.”^10^, ^11^ Then, we convened a diverse group of stakeholders‐including hospital medicine program leaders, faculty development program leaders, and academic administrators at multiple institutions for brainstorming to generate a comprehensive list of potential faculty development topics using existing literature and experiential knowledge. Finally, we pilot tested the tool with two hospital medicine faculty to check for clarity and completeness, as well as to ensure the survey vocabulary matched the target population. The resulting 33 topics ranged from clinical skills enhancement to leadership training and career advancement workshops.

We invited faculty development leaders from seven hospitals. Institutions were selected purposively based on the investigator's convenience and relationship with other institutions. The investigator at the University of Michigan approached the key personnel, such as the hospital medicine division director, at each site to obtain permission to administer the survey and to identify a potential collaborator from their site to help with the study. The site leaders helped identify potential participants at their site and distributed surveys to their faculty via REDCap ® (Research Electronic Data Capture), with invitations sent out via email. Consent was indicated by participants through REDCap before starting the survey. Auto‐reminders were sent weekly via REDCap up to three times if a survey was not completed on time. Data was collected at the University of Michigan from November 2023 to January 2024 and then to the rest of the sites from August 2024 to September 2024.

Survey respondents were asked to review and select from a list of faculty development activities that were important to them and rate each topic on a five‐point scale ranging from critical to not important. Additionally, demographic and professional years of practice data were collected to provide context for the preferences expressed by the participants.

We used descriptive statistics to characterize and quantify faculty requesting each development topic. We then applied LCA to identify unobserved (i.e., latent) classes representing distinct faculty development needs profiles. We included one indicator for each of the 33 faculty development topics variables, dichotomized as “important” or “not important” to maximize interpretability. Applying an exploratory approach without prespecifying the hypothesized number of expected classes, we sequentially derived LCA models containing an increasing number of classes. Selection of the most appropriate latent class model was determined using a combination of the Akaike information criterion (AIC), Bayesian information criterion (BIC) and their adjusted versions (consistent AIC [CAIC] and sample‐size adjusted BIC [SABIC], respectively), probability of class assignment (class separation), class prevalence (favoring models with classes >10% of the sample population), and clinical interpretability.^12^ For each latent class, we assessed the characteristics of the faculty members who were most likely to belong to that class. We also compared the demographic and professional characteristics between the different latent classes using pairwise comparisons to explore potential differences in career stage, institutional roles, and faculty‐specific needs. Analysis was performed using R software.

The study was approved as an exempt/not regulated study by the University of Michigan Institutional Review Board (HUM00244485). This IRB covered participation at all site institutions.

## RESULTS

Of 841 faculty invited, 136 (16.2%) hospitalists from seven academic institutions completed the survey (Table 1). Table 1 shows the number of responses from each institution. The median age of participants was 38 years (interquartile range [IQR], 35–44 years), and the majority (29.4%) had 3–7 years of practice experience. Most faculty (93.4%) identified themselves as career hospitalists, and 54.5% held the rank of Assistant Professor. Over half (51%) of participants spent more than 70% of their time providing direct clinical care. One third of participants reported medical education (33.1%) as their ideal career pathway, followed by leadership (21.3%) and master clinician (19.9%).

**Table 1:** Characteristics of participants.

Characteristic	Overall, *N* = 136
Age, median (interquartile range)	38 (35, 44)
Years practice	*n* (%)
<1	11 (8.1%)
1–3	25 (18.4%)
3–7	40 (29.4%)
7–15	35 (25.7%)
≥15	25 (18.4%)
Time spent in direct care clinical services (without resident coverage)	
<30%	31 (22.8%)
30%–50%	20 (14.7%)
51%–60%	9 (6.6%)
61%–70%	7 (5.1%)
71%–80%	12 (8.8%)
81%–90%	10 (7.4%)
>90%	47 (34.6%)
Career hospitalist	127 (93.4%)
Years of post‐residency	
<1	10 (7.4%)
1–3	21 (15.4%)
3–7	45 (33.1%)
7–15	34 (25.0%)
≥15	26 (19.1%)
Self‐reported Ideal career pathway	
Master clinician	27 (19.9%)
Medical education	45 (33.1%)
Research	16 (11.8%)
Quality improvement	10 (7.4%)
Leadership	29 (21.3%)
Other	9 (6.6%)
Academic rank	
Clinical instructor	19 (14.0%)
Assistant professor	74 (54.4%)
Associate professor	28 (20.6%)
Professor	15 (11.0%)
Survey response by institution	
University of Michigan	56 (41.2%)
Johns Hopkins University	14 (10.3)
Medical College of Wisconsin	8 (5.9%)
University of Colorado	18 (13.2%)
University of Chicago	10 (7.4%)
University of California, San Francisco	18 (13.2%)
University of Texas Southwestern	12 (8.8%)

Table 2 shows the varying levels of importance assigned to different faculty development activities across 33 activities. Overall, the most frequently selected faculty development activities were diagnostic decision‐making (68%), career advancement (66%), leadership development (64%), and resident education skills (64%). In contrast, activities such as workshop development (14.7%), social media skills (15.4%), and procedural skills (19.9%) were less frequently chosen.

**Table 2:** Summary of faculty development activities with perceived importance.

Characteristics	Important, *n* (%)
	(*N* = 136)
Diagnostic decision making	93 (68.4%)
Career advancement	90 (66.2%)
Resident education skill	87 (64.0%)
Leadership development	87 (64.0%)
Giving feedback	80 (58.8%)
Student education skill	77 (56.6%)
Patient safety	77 (56.6%)
Finding joy in medicine	77 (56.6%)
Time management	76 (55.9%)
Teamwork/team building	73 (53.7%)
Scholarly writing	73 (53.7%)
Diversity, equity, and inclusion	72 (52.9%)
Quality improvement	71 (52.2%)
Recognizing and mitigating burnout	67 (49.3%)
Collaboration with other academic institutions	66 (48.5%)
Change management	64 (47.1%)
Effective negotiation	62 (45.6%)
Presentation development	60 (44.1%)
Data analysis	60 (44.1%)
Critically appraising literature	59 (43.4%)
Educational leadership	58 (42.6%)
Data visualization	53 (39.0%)
Public speaking	49 (36.0%)
Curriculum development	44 (32.4%)
Original research	44 (32.4%)
Information management	43 (31.6%)
Policy and advocacy	41 (30.1%)
Systematic reviews	39 (28.7%)
Applying for grants	38 (27.9%)
Case reports	32 (23.5%)
Procedures	27 (19.9%)
Leveraging social media in academic institutions	21 (15.4%)
Workshop development	20 (14.7%)

We investigated models with varying numbers of latent classes and presented the results for models with one to six classes (Table S1). The fit statistics showed that the five‐class model best fits the data as indicated by the lowest values of AIC and SABIC, which are the optimal parameters for LCA with small sample size.^13^ The five‐class model had the strongest interpretation, with representative class proportions ranging from 11% to 29%, and high mean class probabilities (86%–95%).

**Figure 1:**
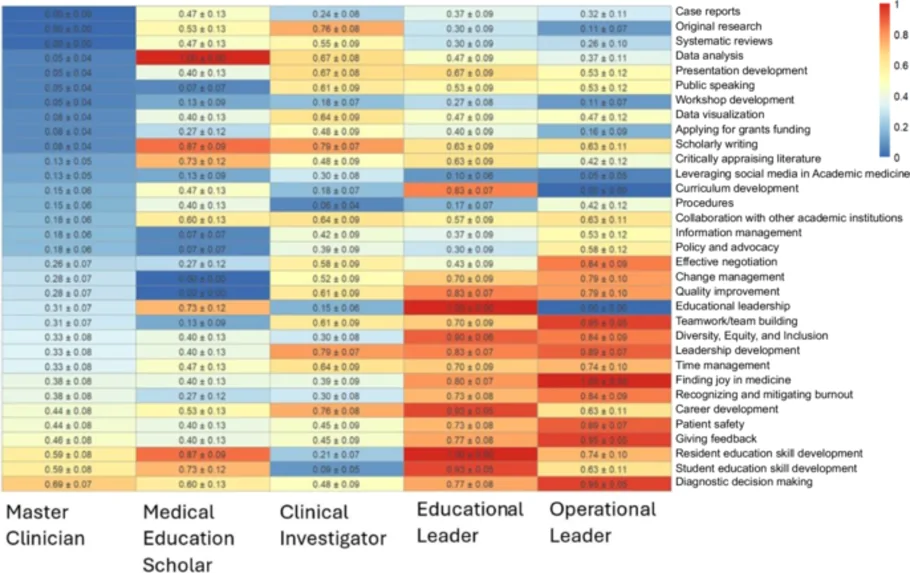
Heatmap displaying the standardized mean values for each variable across groups. *The heatmap shows the proportion of each indicator with the level of “Importance.” 1 indicates “Important” and 0 indicates “Not important.” Data are presented as mean ± SE.

Figure 1 shows the relative proportion of each topic across the different packages. Based on the distribution of these features, we named the five classes as follows:

1. *Master clinician*: This group comprised 29% of the cohort and prioritized activities related to enhancing clinical decision‐making, patient care skills, and clinical excellence.
2. *Clinical investigator* **.** This group comprised 24% of the cohort and was characterized by interest in advancing research and clinical investigation skills, with an emphasis on evidence‐based practice and research methodologies.
3. *Educational leader*: This group comprised 22% of the cohort and sought leadership training within educational contexts, including curriculum design and assessment methodologies.
4. *Operational leader*: This group comprised 14% of the cohort and prioritized leadership skills in hospital administration, with an emphasis on managing operations, quality improvement, and institutional leadership roles.
5. *Medical education scholar* **.** This group comprised 11% of the cohort and focused on improving their educational scholarship, writing, and skills in data analysis.

Table 3 shows differences in faculty characteristics across the development needs profiles. The *master clinician* group had the highest proportion of faculty spending more than 90% of their time on direct clinical service compared to other groups (51.3%). The *medical education scholar* group had the highest proportion of faculty in the lowest experience group (1–<3 years of experience). The *educational leader* group had the most faculty with the academic rank of assistant professor (60.0%). The *clinical investigator* group had the highest proportion of faculty (21.1%) in the most experienced group (i.e., over 15 years of experience), the highest frequency of research‐oriented faculty (33.3%), and the most representation of faculty from a higher academic rank of associate or full professor (48.5%). The *operational leader* group had the highest proportion of faculty pursuing diverse career pathways, such as quality improvement and leadership (26.3% each).

**Table 3:** Characteristics of the five‐class model.

Characteristic	Master clinician	Medical education scholar	Clinical investigator	Educational leader	Operational leader
	39 (28.6%)	15 (11%)	33 (24.3%)	30 (22.1%)	19 (14%)
Age, median (interquartile range)	36 (34, 44)	36 (33, 38)	42 (38, 47)	38 (35, 41)	39 (35, 44)
Years practice, *n* (%)					
<1	2 (5.1%)	1 (6.7%)	2 (6.1%)	4 (13.3%)	2 (10.5%)
1–<3	5 (12.8%)	6 (40.0%)	4 (12.1%)	6 (20.0%)	4 (21.1%)
3–<7	17 (43.6%)	4 (26.7%)	5 (15.2%)	11 (36.7%)	3 (15.8%)
7–<15	7 (17.9%)	3 (20.0%)	11 (33.3%)	8 (26.7%)	6 (31.6%)
≥15	8 (20.5%)	1 (6.7%)	11 (33.3%)	1 (3.3%)	4 (21.1%)
Direct care clinical services, *n* (%)					
<30%	9 (23.1%)	2 (13.3%)	11 (33.3%)	6 (20.0%)	3 (15.8%)
30%–50%	2 (5.1%)	1 (6.7%)	7 (21.2%)	6 (20.0%)	4 (21.1%)
51%–60%	2 (5.1%)	0 (0.0%)	4 (12.1%)	1 (3.3%)	2 (10.5%)
61%–70%	0 (0.0%)	2 (13.3%)	1 (3.0%)	2 (6.7%)	2 (10.5%)
71%–80%	3 (7.7%)	2 (13.3%)	3 (9.1%)	2 (6.7%)	2 (10.5%)
81%–90%	3 (7.7%)	2 (13.3%)	3 (9.1%)	2 (6.7%)	0 (0.0%)
>90%	20 (51.3%)	6 (40.0%)	4 (12.1%)	11 (36.7%)	6 (31.6%)
Career hospitalist, *n* (%)	38 (97.4%)	14 (93.3%)	29 (87.9%)	28 (93.3%)	18 (94.7%)
Ideal career pathway, *n* (%)					
Master clinician	12 (30.8%)	3 (20.0%)	3 (9.1%)	4 (13.3%)	5 (26.3%)
Medical education	18 (46.2%)	6 (40.0%)	2 (6.1%)	18 (60.0%)	1 (5.3%)
Research	0 (0.0%)	4 (26.7%)	11 (33.3%)	1 (3.3%)	0 (0.0%)
Quality improvement	1 (2.6%)	0 (0.0%)	4 (12.1%)	0 (0.0%)	5 (26.3%)
Leadership	6 (15.4%)	2 (13.3%)	11 (33.3%)	5 (16.7%)	5 (26.3%)
Other	2 (5.1%)	0 (0.0%)	2 (6.1%)	2 (6.7%)	3 (15.8%)
Academic rank, *n* (%)					
Clinical instructor	8 (20.5%)	2 (13.3%)	0 (0.0%)	3 (10.0%)	6 (31.6%)
Assistant professor	19 (48.7%)	9 (60.0%)	17 (51.5%)	22 (73.3%)	7 (36.8%)
Associate professor	5 (12.8%)	4 (26.7%)	10 (30.3%)	5 (16.7%)	4 (21.1%)
Professor	7 (17.9%)	0 (0.0%)	6 (18.2%)	0 (0.0%)	2 (10.5%)

## DISCUSSION

Using a novel approach applying LCA to identify and categorize the diverse needs of faculty into distinct faculty development clusters or profiles, we identified five distinct profiles, each with a specific set of preferred activities: master clinician, medical education scholar, clinical investigator, educational leader, and operational leader. These findings align with previously described frameworks^14^, ^15^; however, to our knowledge, this is the first data‐driven classification of faculty development topic preferences in hospital medicine or any other specialty.^2^, ^10^, ^16^, ^17^, ^18^ The study presents an opportunity for a more tailored approach to faculty development, one that aligns development programs with the distinct needs of hospitalists.

Our findings showed that individual faculty needs and interests can be “packaged” for faculty with distinct career pathways. Faculty in the educational and operational leadership profiles showed a strong interest in training related to leadership, mentorship, and non‐technical skills such as team building, negotiation, and time management. Whereas faculty in the clinical investigator and medical education scholar groups sought to develop technical skills, such as research methodologies, writing, and analysis. This emphasizes the need to design tailored development programs that align with each individual faculty member's career pathway. Studies suggest that faculty are more likely to engage in development programs that align with their career goals and interests, which can lead to better outcomes for both the faculty and the institution.^8^, ^19^ Our findings not only identified individual faculty needs but also provided a useful framework for developing targeted development packages that cater to different career stages and interests. For example, a faculty member in the master clinician class may benefit from advanced clinical decision‐making workshops and clinical skill refinement, while a faculty member in the clinical investigator group may require research mentorship and grant writing workshops. Additionally, faculty members in educational leader or operational leader categories may require leadership training that focuses on mentoring, academic leadership, or hospital administration. Although latent class analysis identifies distinct profiles, these should be interpreted as predominant patterns rather than mutually exclusive categories. Overlapping competencies, such as leadership skills, present opportunities to design core faculty development offerings that serve multiple profiles, supplemented by targeted programming for unique needs. Leveraging current offerings through broader professional networks (such as the Society of Hospital Medicine) may further help institutions expand access to development opportunities and strengthen professional communities.

As the profile with the largest membership, the master clinician group deserves special attention. Compared to other groups, faculty members in this class showed less interest in most career development topics, promotions, and advancements, suggesting that these faculty may have nuanced needs that are not represented by the “typical” suite of faculty development programs. This class had the highest proportion of faculty members spending more than 90% of their time on direct clinical services. The heavy demand of patient care, along with challenging schedules and burnout, may limit their ability to participate in existing developmental opportunities. Studies suggest that providing dedicated non‐clinical time could help them invest more time in academic pursuits and prevent burnout.^3^, ^8^, ^18^ Additionally, most faculty in this class are early‐career faculty. Data indicate that the primary drivers of success for early‐career faculty are intrinsic factors related to job satisfaction rather than extrinsic factors such as promotion and academic recognition of their work.^18^ However, the faculty's view may evolve over time as their careers progress and professional identities develop beyond patient care. Studies show that faculty at the mid‐ or late‐career stage often view promotion as recognition for their sustained contributions and leadership.^20^, ^21^ In the later career stages, satisfaction is derived from academic recognition and established professional identity, both within and outside their institution. Therefore, innovative development programs that focus on alignment of such intrinsic and extrinsic factors may better support growth and advancement at all career stages than traditional development offerings.

Our study has several limitations. First, the survey response rate was relatively low (1 in 6), which may introduce response bias. Faculty who are already engaged in development programs may have been more likely to participate, leading to a sample that is not fully representative of the broader hospitalist population. However, the overall distribution of hospitalists respondents by academic rank is representative of the total adult hospitalists in the United States. Second, although we surveyed hospitalists from multiple institutions, the study was limited to academic medical centers and may not fully capture the needs of hospitalists in community or non‐academic settings. Third, given the general scarcity of HM faculty on tenure track, we did not ask respondents to report their academic practice track. Although we did not collect these data, using demographic data for academic HM programs, we can assume many participants in this study are on a clinical promotion track. Finally, the survey focused on preferences for development topics rather than preferred development activities and did not assess how these offerings impact faculty outcomes such as job satisfaction, retention, or career progression. Despite these limitations, the results of this study offer insight into the diverse faculty development needs within hospital medicine and suggest that distinct clusters of faculty members exist with similar development priorities. Future research should explore the long‐term effects of tailored faculty development programs on faculty retention, academic success, promotion, and career fulfillment.

## CONCLUSION

This study presents the first exploration of distinct profiles of faculty development needs in hospital medicine using latent class analysis. By understanding these latent classes of development needs, hospital medicine programs can offer tailored development packages that align with the diverse career paths of their faculty. This approach can lead to more effective faculty development programs that are better aligned with both individual faculty goals and institutional needs, ultimately enhancing faculty satisfaction, retention, and professional growth.

## CONFLICT OF INTEREST STATEMENT

The authors declare no conflicts of interest.

## ETHICS STATEMENT

This study was approved by the University of Michigan Institutional Review Board with exempt/not regulated status (HUM00244485). The authors have relevant financial or non‐financial interests to disclose.

## Supporting information

Supplement Table 1 Fit statistics of latent class models.

Faculty Development Survey.
