# Supplementary material for: Identification of tailored faculty development for diverse career trajectories in hospital medicine
Source: J Hosp Med. 2026 Mar 13;21(9):995–1001. doi: 10.1002/jhm.70292 (PMC13534953; doi:10.1002/jhm.70292)
Supplement: Supplementary file 1 [file JHM-21-995-s001.pdf]

HM Faculty Development Need Assessment

Thank you for your willingness to participate in the Hospital Medicine Faculty Development Assessment Study. The study will help us understand the overall faculty development needs from the faculty's perspective to make changes to the quality of the faculty development program and better shape the future of faculty development. Your participation in this study is completely voluntary. You do not have to participate if you do not wish to. The information collected in this survey will be kept confidential. No identifying information will be shared. The survey will take approximately 5 mins to complete. If you would like to discuss further about the study after completion of this survey, please contact the study PI at [Phone] or via [email]

Before your survey, please read the attached consent form below and click on the link to indicate "Agree" or "Disagree" to participating.

Consent form

[Attachment: "20240603 FacDev_Consent.doc"]

Agree to participate in the study	◯ Yes, I agree to participate<br>◯ No, I do not agree to participate
What is your email address?	__________________________________
What is Your Age?	__________________________________<br>(years)
Name of the institution	__________________________________
How Many Years Have You Been a Practicing Hospitalist?	◯ < 1 year<br>◯ 1-<3 years<br>◯ 3-<7 years<br>◯ 7-<15 years<br>◯ ≥15 years
How much of your professional work is on direct care clinical services?	◯ < 30%<br>◯ 30-50%<br>◯ 51-60%<br>◯ 61-70%<br>◯ 71-80%<br>◯ 81-90%<br>◯ >90%
Do You Consider Yourself a Career Hospitalist?	◯ Yes<br>◯ No

How Many Years Post Residency Are You?	◯ < 1 year<br>◯ 1-<3year<br>◯ 3-<7 years<br>◯ 7-<15 years<br>◯ ≥15 years
How Many Years have you been a Physician, INCLUDING RESIDENCY?	◯ < 1 year<br>◯ 1-<3year<br>◯ 3-<7 years<br>◯ 7-<15 years<br>◯ ≥15 years
Which of the Following Best Describes Your Ideal Career?	◯ Master Clinician (80% or more of your effort dedicated to patient care)<br>◯ Medical Education (>20% of your effort dedicated to education)<br>◯ Research (>20% of your effort dedicated to research)<br>◯ Quality Improvement (>20% of your effort dedicated to quality improvement)<br>◯ Leadership (>20% of your effort dedicated to leadership)<br>◯ Other
If other, please specify.	______________________________
What is Your Academic Rank?	◯ Clinical Instructor<br>◯ Assistant Professor<br>◯ Associate Professor<br>◯ Professor

Which of these Faculty Development Activities are Important to You?

	Critical	Highly Important	Moderately Important	Slightly Important	Not Important
Student Education Skill Development	◯	◯	◯	◯	◯
Resident Education Skill Development	◯	◯	◯	◯	◯
Workshop Development	◯	◯	◯	◯	◯
Public Speaking	◯	◯	◯	◯	◯
Presentation Development	◯	◯	◯	◯	◯
Curriculum Development	◯	◯	◯	◯	◯
Educational Leadership	◯	◯	◯	◯	◯
Giving Feedback	◯	◯	◯	◯	◯
Leadership Development	◯	◯	◯	◯	◯
Effective Negotiating	◯	◯	◯	◯	◯
Policy and Advocacy	◯	◯	◯	◯	◯
Teamwork/Team building	◯	◯	◯	◯	◯

Scholarly Writing	○	○	○	○	○
Data Analysis	○	○	○	○	○
Case Reports	○	○	○	○	○
Systematic Reviews	○	○	○	○	○
Original Research	○	○	○	○	○
Applying for Grant Funding	○	○	○	○	○
Diagnostic Decision Making	○	○	○	○	○
Procedures	○	○	○	○	○
Patient Safety	○	○	○	○	○
Data Visualization	○	○	○	○	○
Critically Appraising Literature	○	○	○	○	○
Time Management	○	○	○	○	○
Change Management	○	○	○	○	○
Quality Improvement	○	○	○	○	○
Information Management	○	○	○	○	○
Career Advancement	○	○	○	○	○
Recognizing and Mitigating Burnout	○	○	○	○	○
Finding Joy in Medicine	○	○	○	○	○
Diversity Equity and Inclusion	○	○	○	○	○
Collaboration with other Academic Institutions	○	○	○	○	○
Leveraging social media in Academic Medicine	○	○	○	○	○

What other Faculty Development activities are important to you?

____
