# Supplementary material for: Identification of tailored faculty development for diverse career trajectories in hospital medicine
Source: J Hosp Med. 2026 Mar 13;21(9):995–1001. doi: 10.1002/jhm.70292 (PMC13534953; doi:10.1002/jhm.70292)
Supplement: Supplementary file 2 [file JHM-21-995-s002.docx]

**Supplementary Table 1. Fit Statistics of Latent Class Models from One to Six Classes**

| **Classes** | **Number of Parameters** | **Smallest Class Size [n (%)]** | **Log Likelihood** | **AIC** | **BIC** | **CAIC** | **SABIC** |
| --- | --- | --- | --- | --- | --- | --- | --- |
| 1 | 1 | 136 (100) | -2877.55 | 5821.10 | 5917.22 | 5830.92 | 5756.85 |
| 2 | 5 | 58 (43) | -2693.70 | 5529.40 | 5736.20 | 5558.88 | 5396.15 |
| 3 | 10 | 37 (27) | -2576.37 | 5370.75 | **5688.23** | **5424.79** | 5170.24 |
| 4 | 15 | 13 (10) | -2595.71 | 5485.41 | 5913.57 | 5564.01 | 5217.65 |
| 5***** | 20 | 15 (11) | -2489.27 | **5348.54** | 5887.38 | 5451.71 | **5013.52** |
| 6 | 25 | 5 (4) | -2561.24 | 5568.48 | 6218.00 | 5696.21 | 5166.21 |

Definition of abbreviations: AIC=Akaike information criterion; BIC=Bayesian information criterion; CAIC= consistent Akaike information criterion; SABIC=sample size–adjusted Bayesian information criterion.

*Chosen model.
